# Cloning and expression analysis of cDNAs corresponding to genes activated in cucumber showing systemic acquired resistance after BTH treatment

**DOI:** 10.1186/1471-2229-4-15

**Published:** 2004-08-26

**Authors:** Catherine Bovie, Marc Ongena, Philippe Thonart, Jacques Dommes

**Affiliations:** 1 Laboratoire de Biologie Moléculaire et de Biotechnologie Végétales, Département des Sciences de la Vie, B22, Université de Liège, B-4000 Liège/Sart Tilman, Belgium; 2 Centre Wallon de Biologie Industrielle, Unité de Bioindustries, Faculté Universitaire des Sciences Agronomiques, B-5030 Gembloux, Belgium

## Abstract

**Background:**

Infection of plants by necrotizing pathogens can lead to the rapid and localized induction of a complex set of defense responses resulting in a restriction of pathogen growth and spread. Subsequently, an increase of plant resistance against a broad spectrum of pathogens is observed systemically. This plant immunity is known as Systemic Acquired Resistance. To identify components of the transduction pathway, we cloned and analysed the expression pattern of several mRNAs accumulating in cucumber plants after induction of Systemic Acquired Resistance.

**Results:**

We tested on cucumber different compounds known to induce systemic acquired resistance. Among these, BTH (benzo(1,2,3)thiadiazole-7-carbothioic acid S-methyl ester) proved to be very effective. mRNA RT-PCR differential display was used to identify mRNA sequences induced 24 hours after the application of 10 μM BTH to cucumber plants. A cDNA library constructed from cucumber plants sprayed with 10 μM BTH was screened to get corresponding full length cDNAs. Among the identified cDNAs were those coding for a putative ras-related GTP-binding protein, a putative beta-1,4-*N*-Acetylglucosaminyltranferase III and a putative pathogenesis related protein. The time course of accumulation of the three corresponding mRNAs was analysed by northern blotting in plants treated by BTH or in plants infected by *Colletotrichum lagenarium*.

**Conclusions:**

The mRNA RT-PCR differential display technique allowed the identification of three genes possibly involved in Systemic Acquired Resistance in cucumber. Pathogenesis-related proteins are known to be involved in plant defence against pathogens. GTP-binding protein and *N*-acetylglucosaminyltranferase III have been reported to be components of signal transduction pathways in mammals and plants.

## Background

Infection of plants by necrotizing pathogens can lead to the induction of a complex set of defense responses resulting in a restriction of pathogen growth and spread. The infected leaves develop a hypersensitive response (HR), i.e., rapid, localized cell death occurring at the sites of pathogen entry [1]. Concomitant with the HR is the accumulation of salicylic acid and several classes of pathogenesis-related (PR) proteins, many of which exhibiting antimicrobial activity [2]. Subsequently, an enhancement of the plant defensive capacity against a broad spectrum of pathogens is observed. This resistance is expressed locally as well as in distal, uninfected tissues and can last for several weeks to months. It is known as Systemic Acquired Resistance or SAR [3].

Salicylic acid was identified as an important signal in the SAR transduction pathway. It was shown to accumulate during the onset of SAR in cucumber [4], tobacco [5], and *Arabidopsis thaliana *[6]. Exogenously supplied salicylic acid induces the same set of genes and resistance against the same spectrum of pathogens, as with pathogen-induced SAR [3]. Plants in which salicylic acid accumulation is prevented by over expression of a bacterial salicylate hydroxylase gene failed to develop SAR and/or exhibited increased susceptibility to pathogen infection [7,8].

Analysis of *A. thaliana *mutants has revealed that the NPR1 protein (also known as NIM1) is required for SAR induction and acts downstream of salicylic acid in the SAR pathway. Plants carrying mutations in this gene fail to express several *PR *genes and display enhanced susceptibility to infection [9,10]. The NPR1/NIM1 protein interacts with members of the TGA/OBF family of basic leucine zipper transcription factors. Some of these factors have been shown to bind to salicylic acid-responsive promoter elements of the *PR-1 *gene [11-13]. The salicylic acid induced interaction of NPR1 with TGA factors is localized in the nucleus [14], while cytoplasmic NPR1 appears to modulate crosstalk between salicylic acid- and jasmonate-dependent defense pathways [15]. Other components of the salicylic acid signaling pathway have been identified using genetic approaches in *Arabidopsis thaliana *[16]. They include PAD4 and EDS3 which activate EDS4, SID2 and EDS5 leading to salicylic acid accumulation [17].

Besides pathogens, SAR is also induced by exogenously applied chemicals. In addition to salicylic acid, well-known chemical inducers of *PR *genes include 2,6-dichloroisonicotinic acid (INA), benzo(1,2,3)thiadiazole-7-carbothioic acid S-methyl ester (BTH) and L-α-amino butyric acid (ABA) [18-23]. SAR induction appears to be governed by a complex signal transduction process [24] that involves a signal originating at the site of infection or treatment, and moving throughout the plant. Chemical inducers provide tools for dissection of the signal transduction pathway that regulates the defense responses (they enter the pathway at different points). On another hand, the cucumber plant provides a good model system for studying induced disease resistance [25]. Local and systemic increases in chitinase and/or peroxidase activity have been observed in response to inoculation with necrotizing pathogens or to treatment with SA, INA and BTH [26-28].

The aim of the present work was to identify and clone mRNAs accumulating after induction of SAR in cucumber plants. These sequences could correspond to proteins potentially involved in the signal transduction pathway leading to SAR or to proteins responsible for the state of resistance. In a first set of experiments, potential SAR inducing chemicals were tested in order to set up a reproducible system of activation of this resistance in cucumber. The mRNA RT-PCR differential display method [29] was then used to identify genes activated during SAR induction. Several differentially expressed cDNA were identified and some of them were cloned and sequenced. Their expression was analyzed by Northern blotting.

## Results

### Chemically induced resistance in cucumber

The SAR inducing activity of different chemicals has already been documented in one or several plant species [18-23]. To evaluate their activity on cucumber, cotyledons of two-week-old plants were infiltrated with 0.5 mM salicylic acid, 50 mM L-α-amino butyric acid, 0.78 mM 2-thiouracil, 1 mM thiamine or 20 mM BaCl_2_. Control plants were infiltrated with water. In a parallel experiment, four-week-old cucumber plants (with two true leaves) were sprayed with 5 mM salicylic acid, 50 mM L-α-amino butyric acid, 0.78 mM 2-thiouracil, 1 mM thiamine, 20 mM BaCl_2 _or 1 mM BTH. Control plants were sprayed with water or left untreated. Total RNAs were extracted from treated leaves 6 h, 24 h or 48 h after treatment. Induction of *PR-8 *gene was assayed by Northern blotting followed by hybridization with a cucumber PR-8 cDNA probe. *PR-8 *cucumber gene encodes an acidic chitinase which accumulates after tobacco necrosis virus (TNV) infection or salicylic acid induction and is considered as a SAR molecular marker [30].

In infiltration experiments (results not shown), control plants showed positive hybridization signals. To check whether this activation of the *PR-8 *gene could be due to wounding during infiltration, the effect of wounding alone was evaluated by making a cut from the midvein to the outer edge of the cotyledons. A strong signal was obtained after wounding which could be explained by induction of the acidic chitinase itself or of an isoform. Therefore infiltration did not prove to be an appropriate method for applying SAR inducers in cucumber, because it probably induced a wound response.

**Figure 1:**
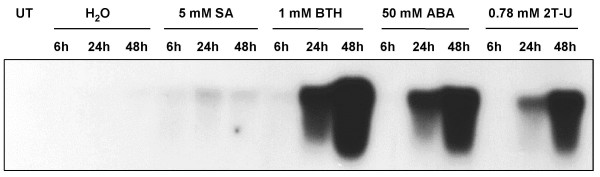
**Time course of PR-8 mRNA accumulation in cucumber leaves in response to chemical treatments. **Total RNA was extracted from cucumber leaves at various time after spraying with water, 5 mM salicylic acid, 1 mM BTH, 50 mM L-α-amino butyric acid (ABA), 0.78 mM 2-thiouracil (2T-U) or from untreated leaves (UT). RNA (10 μg) was fractionated by electrophoresis on agarose gel. Northern blot was probed with α-^32^P labeled PR-8 cDNA (loading of equal amounts of RNA was confirmed by ethidium bromide staining).

When applied by spraying, BTH, L-α-amino butyric acid and 2-thiouracil strongly induced PR-8 mRNA accumulation, while SA treatment led to a lower induction level (Fig. 1). Thiamine and BaCl_2 _induced slightly *PR-8 *expression (results not shown). RNA from control plants did not show any hybridization signal indicating that spraying itself did not induce *PR-8 *expression. BTH, which is an analogue of salicylic acid, showed the strongest *PR-8 *induction. But cucumber plants treated with 1 mM BTH showed a reduced growth (smaller leaves area and shorter internodes). As BTH was shown to be a very efficient activator of disease resistance in different plant species [20-23], we tried to induce SAR in cucumber plants with lower BTH concentrations (10 μM, 30 μM and 100 μM). PR-8 transcript accumulation was quite similar for the three concentrations (see Additional file 1) and no adverse effect on plant growth was observed.

**Figure 2:**
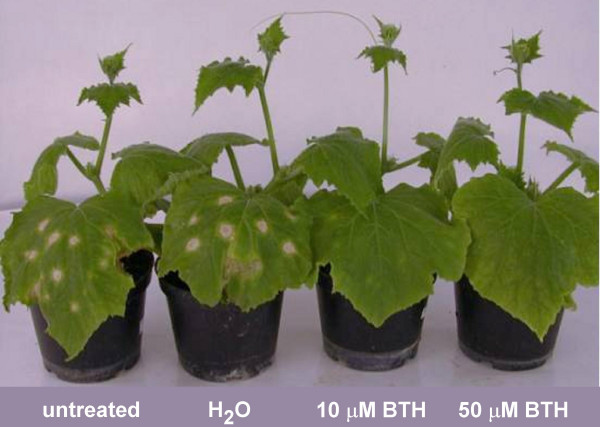
**Local acquired resistance induced by 10 μM or 50 μM BTH. **Four-week-old cucumber plants (with two true fully expanded leaves) were sprayed with H_2_O, 10 μM BTH, 50 μM BTH or left untreated. The second true leaf was infected with *C. lagenarium *5 days later. Plants were photographed 2 weeks postinoculation.

In cucumber, SAR has been shown to be effective against anthracnose caused by *Colletotrichum lagenarium *and salicylic acid treatment was shown to induce resistance against this pathogen [31]. To confirm that BTH acts as a chemical inducer of SAR in cucumber, four-week-old plants were sprayed with water, 10 μM BTH, 50 μM BTH, or left untreated, 5 days prior to inoculation of the second true leaf with *C. lagenarium*. Two weeks after inoculation, the plants were scored for spreading lesions, i.e. lesions showing a larger size than the initial inoculated area. As shown in figure 2 and table 1, BTH treatment resulted in decreased symptoms. Control leaves inoculated with *C. lagenarium *showed large spreading lesions and thus extensive fungal growth. Treatment of plants with BTH prior to *C. lagenarium *inoculation resulted in a markedly decreased lesion formation (Fig. 2). In untreated plants and water treated plants, 67 and 64 % of inoculation sites showed spreading lesions respectively (Table 1). In BTH treated plants, only 23 % (10 μM BTH) or 9 % (50 μM BTH) of inoculated sites developed spreading lesions (Table 1).

To check whether BTH is able to act systemically or is transported through cucumber plants, the third leaf of four-week-old plants was wrapped with a plastic film and the plants were sprayed with H_2_O, 10 μM BTH, 50 μM BTH or left untreated. This leaf was inoculated with *C. lagenarium *7 days later. The percentage of spreading lesions on third leaves from pathogen-challenged untreated or H_2_O treated cucumber plants (infected controls) were respectively 86% and 83.5%. In BTH treated plants, 38 % (10 μM BTH) or 12 % (50 μM BTH) of inoculated sites developed spreading lesions (Table 2). These results suggest that BTH can activate resistance in untreated leaves.

### Differential expression of genes in BTH induced cucumber plants

**Figure 3:**
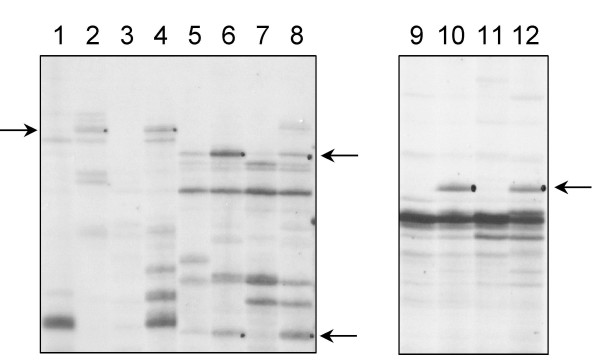
**Zoom in on an auto-radiogram of differentially displayed RT-PCR products. **The RT-PCR were performed with primers 5'T_11_GA and 5'GATCTAACCG (lane 1 to 4), primers 5'T_11_GA and 5'GATCAATCGC (lane 5 to 8), primers 5'T_11_GA and 5'TACAACGAGG (lanes 9 to 12). Uneven lanes: H_2_O treated plants. Even lanes: 10 μM BTH treated plants. The arrows show bands corresponding to transcripts accumulating only or presenting a higher expression in BTH treated plants.

**Figure 4:**
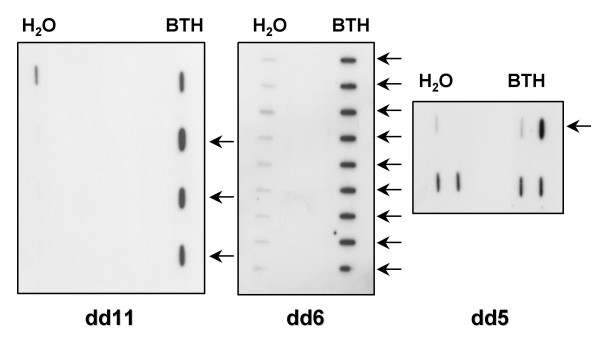
**Slot blotting of PCR amplified fragments identified in differential display experiments. **PCR amplified DNA from four dd11 clones, nine dd6 clones and four dd5 clones respectively were hybridized with the ^32^P labeled RT-PCR corresponding products from either H_2_O or BTH treated plants. The arrows show DNA from clones containing differentially displayed fragments.

Plants were sprayed with 10 μM BTH or water. Sampling was performed 24 h after treatment in order to point out early-induced differences in gene expression. RT-PCR products were separated on polyacrylamide gels. Bands that appeared on the display of RNA only from BTH treated plants, or only from water treated plants were likely to correspond to differentially expressed mRNAs. Twelve differentially expressed bands were identified (Table 3). Their sizes ranged between 250 and 450 bp. They corresponded to genes expressed in the BTH treated plants and not in the control ones or presenting a higher expression in the BTH treated plants compared to the control ones (Fig. 3). These bands were eluted from the gel, PCR amplified and cloned in the pGEM-T vector. In order to confirm the differential expression of these genes, reverse slot blot hybridizations were performed: DNA from four to ten clones obtained from each differentially displayed band was fixed on a membrane and hybridized with the corresponding reverse transcribed products from either BTH or H_2_O treated plants (Fig. 4). Six from the twelve fragments were confirmed to be differentially expressed: dd3, dd4, dd5, dd6, dd7 and dd11. Five bands (dd1, dd8, dd9, dd10 and dd12) were either expressed evenly between BTH and H_2_O treated plants (differential display false positives or signal masked by the expression of homologous genes). In the remaining case (dd2), no signal could be observed on the slot blots. This absence of hybridization signal could be due to a lack of sensitivity of the slot blot hybridization procedure.

The dd3, dd4, dd5, dd6, dd7 and dd11 cDNA fragments were sequenced. Blast analysis did not lead to identification of any homology with known sequences. This is not surprising as mRNA differential display generates fragments corresponding to 3'untranslated regions for which homologies are difficult to find. Two of the six fragments were proved to be part of the same gene (dd5 and dd7). A cDNA library was constructed from cucumber plants sprayed with 10 μM BTH (leaves were sampled 24 h after treatment). The library was screened with the five differentially expressed fragments. Hits were obtained for four of these probes (corresponding to dd3, dd4, dd6 and dd11). Full length cDNAs corresponding to dd4, dd6 and dd11 were obtained. Homology between cloned cDNA and sequences in databases is presented in table 4. The cDNA dd3 matched to a putative RAS-related GTP-binding protein from *A. thaliana*. It will be referred to as CRG (cucumber RAS-related GTP-binding protein). The cDNA dd4 matched to a translation releasing factor 2. It will be referred to as CTR (cucumber translation releasing factor 2). The cDNA dd6 matched to a putative β-1,4-*N*-Acetylglucosaminyltranferase III from *A. thaliana*. This protein was named CGT (cucumber acetylglucosaminyltransferase III). The cDNA dd11 matched to a putative pathogenesis related protein from *A. thaliana *and, with slightly less homology, to a pathogenesis related protein from barley induced by fungal infection [32]. It will be referred to as CPR (cucumber pathogenesis-related protein).

### *PR-8*, *CPR*, *CRG and CGT *expression analysis

**Figure 5:**
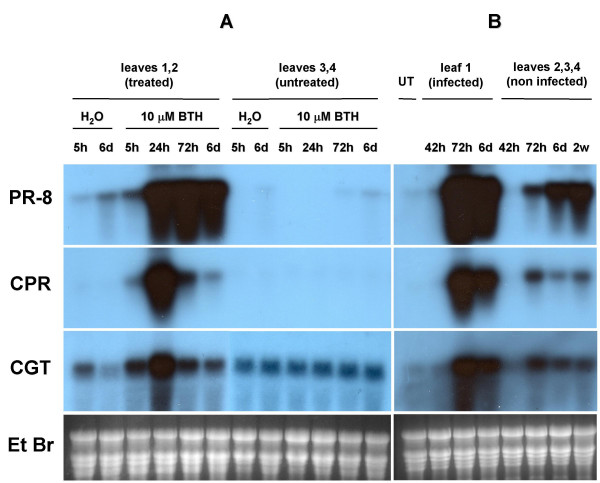
**Time course of mRNA accumulation in cucumber leaves after BTH treatment (A) or *C. lagenarium *inoculation (B). **A: Total RNA was extracted from cucumber leaves at various time after application of 10 μM BTH or water (mock treatment). RNA (15 μg) from leaves 1 and 2 (treated) and leaves 3 and 4 (untreated) was fractionated by electrophoresis on agarose gels. Identical Northern blots were probed with ^32^P-labeled PR-8, CPR and CGT cDNAs. B: Total RNA was extracted from cucumber leaves at various time after *C. lagenarium *inoculation. RNA (15 μg) from untreated leaves (UT), leaves 1 (infected) and leaves 2, 3 and 4 (non infected) was fractionated by electrophoresis on agarose gels. Identical Northern blots were probed with ^32^P-labeled PR-8, CPR and CGT cDNAs. Equal loading of RNA was confirmed by ethidium bromide (Et Br) staining. A representative gel is shown.

To investigate the expression of *PR-8*, *CPR*, *CRG *and *CGT *genes in cucumber, the two first leaves of four-week old plants were sprayed with 10 μM BTH. In another batch of plants, the first leaf was inoculated with *C. lagenarium*. Total RNA was extracted from treated leaves or upper untreated leaves at various times after treatment. Accumulation of the PR-8, CPR, CRG and CGT mRNAs was assayed by Northern blotting followed by hybridization with the corresponding cDNA probes (fig. 5).

Expression of *PR-8 *gene was induced at a high level by both 10 μM BTH spraying and *C. lagenarium *infection in the leaves submitted to the treatment. PR-8 mRNA could be detected as soon as 5 h after BTH application and a maximum of expression could be observed after 72 h. *PR-8 *expression was observed as from 72 h after inoculation with *C. lagenarium*. No significant PR-8 mRNA accumulation could be detected in the untreated leaves from BTH sprayed plants, while in non infected leaves *PR-8 *expression was observed 72 h after infection by *C. lagenarium*; the level of expression was still increasing 2 weeks after *C. lagenarium *inoculation.

*CPR *gene expression was strongly induced in both 10 μM BTH treated and *C. lagenarium *inoculated leaves. CPR mRNA could be detected as soon as 5 h after BTH application and a maximum of expression could be observed after 24 h. *CPR *expression was observed 72 h after infection by *C. lagenarium*. No expression of *CPR *gene could be observed in untreated leaves from BTH sprayed plants, while CPR mRNA accumulation could be detected in uninfected leaves from *C. lagenarium *infected plants 72 h after inoculation.

*CGT *gene showed a significant basal level of expression as it could be observed in control plants (fig. 5). Nevertheless the expression of *CGT *was increased both by spraying with 10 μM BTH (with a maximum at 24 h) and by inoculation with *C. lagenarium *(with a maximum at 72 h) in the leaves submitted to the treatment. A systemic induction could also be observed in untreated leaves from *C. lagenarium *inoculated plants (as from 72 h after infection).

The expression level of the *CRG *gene was too low to allow us to show an induction of the expression (data not shown).

Expression of the same genes was analyzed in wounded cucumber plants. A cut was made from the midvein to the outer edge of the first leaf. Total RNA was extracted from the cut leaf or the upper leaf, 24 and 48 h after wounding. An induction of the expression of *PR-8 *and *CPR *genes was only observed in the cut leaf 24 h after wounding. No expression was observed later nor in the uncut upper leaf. The *CGT *and *CRG *genes were not induced by wounding (results not shown).

Expression of these genes was also analyzed in cucumber plants showing rhizobacteria-induced systemic resistance. Plants were grown in compost drenched with a *Pseudomonas putida *BTP1 suspension, a plant growth-promoting rhizobacteria known to promote induced systemic resistance in cucumber [33]. Total RNA was extracted from cotyledons collected three weeks after sowing. *PR-8*, *CPR*, *CGT *and *CRG *did not show any induction of expression in these conditions (results not shown).

## Discussion

We tested here two different techniques and several compounds to induce SAR in cucumber. The expression of *PR-8 *gene, which encodes an acidic chitinase from cucumber, was used as an indicator of the activation of the defense response. The accumulation of this acidic chitinase after tobacco necrosis virus infection or salicylic acid induction correlates with induced resistance [30]. Among the different compounds tested, BTH applied by spraying proved to be the stronger inducer of PR-8 mRNA accumulation. BTH sprayed at a concentration of 10 μM was still able to induce *PR-8 *gene expression. The protective effects of BTH on cucumber were confirmed in challenge experiments with *C. lagenarium*. Spraying cucumber plants with BTH (10 μM or 50 μM) allowed effective local and systemic protection against *C. lagenarium*. BTH was recently identified as a safe, reliable and non phytotoxic plant protection agent by scientists at Novartis. Exogenous application of BTH to tobacco, *A. thaliana*, wheat and cucumber has been shown to activate a number of SAR associated genes leading to enhanced plant protection against various pathogens [20-22,28].

Identifying SAR induced genes could provide clues to elucidate the signal transduction pathway leading to plant defense responses. We used RT-PCR differential display in BTH-treated cucumber plants to detect SAR associated mRNAs. We have identified four BTH-inducible genes: a putative ras-related GTP binding protein (CRG), a putative translation releasing factor 2 (CTR), a putative β-1,4-*N*-acetylglucosaminyltranferase III (CGT) and a putative pathogenesis related protein (CPR). Expression of *PR-8*, *CPR *and *CGT *genes was shown to be induced by 10 μM BTH treatment as well as *C. lagenarium *infection. The response to BTH was observed earlier (5 h after treatment) than the one developed after pathogen inoculation (72 h after infection). However, systemic expression of these genes was only observed in *C. lagenarium *infected plants.

Small GTP-binding proteins form a large family of nucleotide triphosphatases whose activity is related to the binding, hydrolysis and release of guanosine triphosphate [34]. MAP kinase cascade represents an important downstream effector pathway for RAS in most cells [35] and recent studies have shown that MAP kinases are activated in plants in response to pathogen attack and wounding [36,37]. Small GTP-binding proteins have been shown to be involved in important cell mechanisms like cell division, transduction of external signals across the plasma membrane, endocytosis and exocytosis, cell death in plants and establishment of plant defense reaction [34,38-42]. The β-1,4-*N*-Acetylglucosaminyltranferase III catalyses the addition of *N*-acetylglucosamine to the β-mannoside of the tri-mannose core in *N*-glycans, resulting in the suppression of further processing and elongation. In mammals, *N*-acetylglucosaminyltransferase III expression is associated with differentiation, cell adhesion and tumor progression [43,44]. It was shown recently to interfere with epidermal growth factor signaling and H_2_O_2_-induced activation of protein kinase C [45,46]. *N*-linked glycans were shown to be widely distributed in plants and highly expressed at the cell surface, which might suggest a putative function in cell/cell communication [47]. To our knowledge β-1,4-*N*-Acetylglucosaminyltranferase has never been described in the context of a plant-pathogen interaction. Several cDNAs or gene encoding enzymes involved in the biosynthesis of complex *N*-linked glycans have been cloned from plants. They include *N*-acetylglucosaminyltranferase I cDNAs from *A. thaliana*, potato and tobacco [48], *N*-acetylglucosaminyltransferase II cDNA from *A. thaliana *[49] and defense-related glucosyl transferase gene from tomato [50]. GTP-binding proteins and *N*-acetylglucosaminyltranferases are involved in important mechanisms, notably signaling pathways and therefore seem of high interest. The putative PR protein, which has been first identified in barley after fungal infection [32] has no known function.

Only *PR-8 *and *CPR *genes were induced by wounding but not systemically. *PR-8 *and *CPR *genes were not induced by *P. putida *BTP1 treatment, which triggers induced systemic resistance (ISR) in cucumber. ISR is phenotypically similar to SAR but the mechanisms of this resistance were shown to be different: although this was not tested with *P. putida *BTP1, in other experimental systems ISR was shown to be dependent on NPR/NIM1 function, but does not involve salicylic acid, nor PR protein accumulation [51]. Our results correlate with those of Pieterse et al. who showed that induced systemic resistance in *A. thaliana *was independent of *PR *genes expression [51]. In the same way, ISR also seems to be independent on *CGT *and *CRG *expression, as these genes were not induced by *P. putida *BTP1.

## Conclusions

Among different SAR inducers tested on cucumber, BTH proved to be very efficient. Applied by spraying, BTH induced PR-8 mRNA accumulation and allowed effective local and systemic protection against *C. lagenarium*. Using RT-PCR differential display, we identified four BTH-inducible genes, including a putative ras-related GTP binding protein (CRG), a translation releasing factor 2 (CTR), a putative β-1,4-*N*-acetylglucosaminyltranferase III (CGT) and a putative pathogenesis related protein (CPR). Genes expression studies confirmed the local induction of *CGT *and *CPR *after BTH treatment or *C. lagenarium *infection and their systemic induction in response to *C. lagenarium *infection. Moreover *CPR *was locally induced by wounding. The significant level of expression of *CGT *and *CPR *genes during defense responses suggests a potential role for the gene products in the SAR pathway or in the state of resistance in cucumber.

## Methods

### Organisms and growth conditions

Cucumber plants (*Cucumis sativus *cv. *Marketer*) were grown in compost at 22°C with a 16 h light photoperiod.

Cultures of *Colletotrichum lagenarium *were maintained on malt agar at 25 °C. Spores suspensions in water supplemented with 0.01 % tween 80 were prepared from four- to six-week-old cultures grown in petri plates. Suspensions were filtered through cheesecloth and the spore concentration determined with a hemacytometer.

### Chemical treatments

All chemicals were obtained from Sigma except for BTH (provided by Novartis). Chemicals were dissolved in water and the pH adjusted to 6.6 with NaOH except for thiamine, BaCl_2 _and BTH. Infiltration experiments: cotyledons of two-week-old plants were infiltrated through a vein until the whole leaf was impregnated with the solution. Spraying experiments: the two first true leaves of four-week-old cucumber plants were sprayed with +/- 3 ml solution/plant. The upper leaves were protected with a plastic sheet when systemic resistance was to be tested.

### ISR induction

Prior to planting, disinfected cucumber seeds were soaked for 10 min in a *Pseudomonas putida *BTP1 suspension (4.10^8 ^CFU/ml in 0.85% NaCl). Control seeds were soaked in 0.85% NaCl. Cucumber seeds were sown in 10 cm-pots containing sterilized potting soil previously mixed with a *P. putida *BTP1 inoculum to a final concentration of 3.10^7 ^CFU/g or with an equal volume of sterile water for control plants. Cucumber were germinated at 25°C with a 16 h light photoperiod. Six and 12 days after sowing, 20 ml of a bacterial suspension at 10^8 ^CFU/ml were added as a drench to the roots. Control plants were watered with 20 ml of 0.85% NaCl.

### *C. lagenarium *infection

Plants were placed in a humidity chamber 48 hours before infection experiments. The first true leaf was inoculated with 10 drops (10 μl) of a conidial suspension of *C. lagenarium *(10^6 ^spores/ml). In challenge experiments, inoculation was performed 5 (or 7) days after chemical treatment on the second or third leaf.

### Plant RNA extraction and analysis

The extraction of total RNA from cucumber leaves (5 plants per treatment) was performed using a phenol/SDS method [52]. Northern hybridization of RNA fractionated by agarose-formaldehyde gel electrophoresis [52] was performed with a α-^32^P PR-8, CPR, CGT or CRG cDNA probe (Random Primed DNA labeling kit, Roche).

### mRNA Differential Display

The mRNA differential display was performed using the differential display kit from Eurogentec (Belgium) and following the instructions of the supplier. PCR products were labeled with α-^35^S dATP, separated by electrophoresis on denaturing 6% polyacrylamide gels and visualized by autoradiography. PCR reactions showing differentially displayed bands were repeated twice on two different RNA samples in order to reduce the number of false-positives.

### Cloning and sequencing

Differentially displayed bands were excised and eluted during 30 min in 100 μl of H_2_O followed by boiling during 10 min, then precipitated and suspended in 10 μl of H_2_O. A second PCR amplification was performed on 4 μl of DNA following the same protocol than for the differential display itself. Products were cloned in pGEM-T vector (Promega) and manually sequenced by the dideoxy sequencing method. Available public databases (i.e. EMBL) were searched for homology with our sequences using the GCG software package (Blastall, Fasta).

### Slot-Blot hybridisation

PCR amplified DNA from four to ten clones of each differentially displayed band was blotted on a positively charged nylon membrane using a filtration manifold and following the instructions of the supplier (Hoefer). Blots were hybridized with the ^32^P labeled RT-PCR corresponding products from either BTH or H_2_O treated plants.

### cDNA library construction and screening

mRNA from cucumber plants sprayed with 10 μM BTH (leaves were sampled 24 h after treatment) were purified with the PolyATractmRNA Isolation System III from Promega. The cDNA library was performed with the SMART cDNA Library Construction Kit from Clontech following the instructions of the supplier for cDNA synthesis by primer extension. The library was screened with ^32^P labeled cDNA probes. cDNA sequencing was performed at Genome Express, France. Available public databases (i.e. EMBL) were searched for homology with our sequences using the GCG software package (Blastall, Fasta).

## Authors' contributions

CB carried out the plants treatments, the molecular biology studies and the database research and drafted the manuscript. MO supplied the pathogen and participated in the challenge and ISR induction experiments. JD and PT participated in the design and coordination of the study. All authors read and approved the manuscript.

## Supplementary Material

Additional File 1 **PR-8 mRNA accumulation in cucumber leaves in response to different concentrations of BTH**. Total RNA was extracted from cucumber leaves 24 h and 48 h after spraying with water, 10 μM BTH, 30 μM BTH, 100 μM BTH or from untreated leaves (UT). RNA was fractionated by electrophoresis on agarose gel. Northern blot was probed with α-^32^P labeled PR-8 cDNA. Loading of equal amounts of RNA was confirmed by ethidium bromide (Et Br) staining. Click here for file

## Figures and Tables

**Table 1:** Local acquired resistance induced by 10 μM or 50 μM BTH.

	untreated	H_2_O	10 μM BTH	50 μM BTH
Exp.1	66 ± 24 %	57 ± 25 %	12 ± 9 %	13 ± 11 %
Exp.2	68 ± 28 %	71 ± 23 %	34 ± 30 %	5 ± 6 %
Mean value	67 %	64 %	23 %	9 %

Four-week-old cucumber plants (with two true fully expanded leaves) were sprayed with H_2_O, 10 μM BTH, 50 μM BTH or left untreated. The second true leaf was infected with *C. lagenarium *5 days later (ten infection sites per leaf). The percentages of spreading lesions (i.e. lesions showing a larger size than the initial inoculated area) observed 2 weeks after inoculation with *C. lagenarium *were calculated on the basis of 14 plants/treatment (experiment 1) and 16 plants/treatment (experiment 2) (means +/- standard deviations).

**Table 2:** Systemic acquired resistance induced by 10 μM or 50 μM BTH.

	untreated	H_2_O	10 μM BTH	50 μM BTH
Exp.1	76 ± 20 %	69 ± 17 %	37 ± 19 %	18 ± 16 %
Exp.2	96 ± 6 %	98 ± 3 %	39 ± 15 %	7 ± 9 %
Mean value	86 %	83.5 %	38 %	12,5 %

Four-week-old cucumber plants (with two true fully expanded leaves) were sprayed with H_2_O, 10 μM BTH, 50 μM BTH or left untreated. The third true leaf was infected with *C. lagenarium *7 days later (ten infection sites per leaf). The percentages of spreading lesions (i.e. lesions showing a larger size than the initial inoculated area) observed 2 weeks after inoculation with *C. lagenarium *were calculated on the basis of 18 plants/treatment (experiment 1) and 17 plants/treatment (experiment 2) (means +/- standard deviations).

**Table 3:** Oligonucleotide primers used in RT-PCR that allowed to amplify the differentially displayed cDNA fragments.

Clone name	Downstream primer used	Upstream primer used
dd1	5' T_11_AA	5' CTGCTTGATG
dd2	5' T_11_CG	5' GATCAATCGC
dd3	5' T_11_CG	5' GATCAATCGC
dd4	5' T_11_CG	5' TCGGTCATAG
dd5	5' T_11_GA	5' AAACTCCGTC
dd6	5' T_11_GA	5' TTTTGGCTCC
dd7	5' T_11_GA	5' GATCTAACCG
dd8	5' T_11_GA	5' GATCAATCGC
dd9	5' T_11_GA	5' GATCAATCGC
dd10	5' T_11_GA	5' TCGATACAGG
dd11	5' T_11_GA	5' TACAACGAGG
dd12	5' T_11_GG	5' TACAACGAGG

**Table 4:** Characteristics of isolated cDNA clones.

Clone name	Clone length (bp)	Accession N°	Matching sequence from data base	Origin of matching sequence and accession number	Amino acid sequence % identity (*)
dd3	774	AJ634908	Ras-related GTP-binding protein	*A. thaliana *NP 177505	65 % (1e^-73^)
dd4	1533	AJ635428	Translation releasing factor 2	*A. thaliana *NP 851097	70 % (7e^-170^)
dd6	2192	AJ629867	β-1,4-*N*-acetylglucosaminyltranferase	*A. thaliana *NP 172759	77 % (0.0)
dd11	1588	AJ629866	Pathogenesis-related protein	*A. thaliana* NP 565189	51 % (1e^-51^)

* expected value of best match
