# Supplementary figures and images for: Cloning and expression analysis of cDNAs corresponding to genes activated in cucumber showing systemic acquired resistance after BTH treatment
Source: BMC Plant Biol. 2004 Aug 26;4:15. doi: 10.1186/1471-2229-4-15 (PMC516775; doi:10.1186/1471-2229-4-15)

## Slide 1
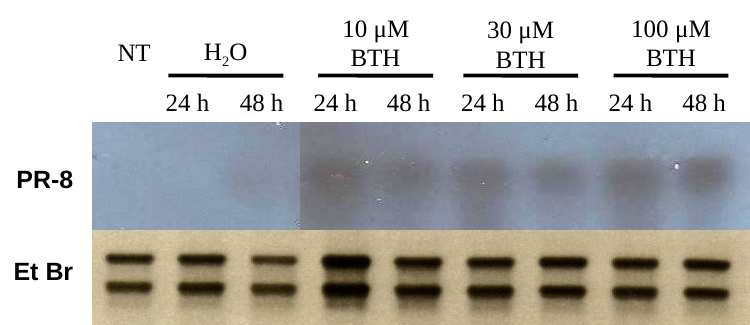

10 μM
BTH
100 μM
BTH
30 μM
BTH
H2O
NT
24 h
48 h
24 h
48 h
24 h
48 h
24 h
48 h
PR-8
Et Br

Supplement: Additional File 1 — PR-8 mRNA accumulation in cucumber leaves in response to different concentrations of BTH. Total RNA was extracted from cucumber leaves 24 h and 48 h after spraying with water, 10 μM BTH, 30 μM BTH, 100 μM BTH or from untreated leaves (UT). RNA was fractionated by electrophoresis on agarose gel. Northern blot was probed with α-32P labeled PR-8 cDNA. Loading of equal amounts of RNA was confirmed by ethidium bromide (Et Br) staining. [file 1471-2229-4-15-S1.ppt]
